# Control glucémico y estudio del metabolismo lipídico y del óseo en niños con diabetes de tipo 1

**DOI:** 10.7705/biomedica.7132

**Published:** 2024-05-31

**Authors:** Pilar Calmarza, Rasha Isabel Pérez-Ajami, Carlos Prieto-López, Alba Gallego-Royo, Celia García-Carro, Graciela María Lou-Francés

**Affiliations:** 1 Servicio de Bioquímica Clínica, Hospital Universitario Miguel Servet, Zaragoza, España Hospital Universitario Miguel Servet Hospital Universitario Miguel Servet Zaragoza Spain; 2 Instituto de Investigación Sanitaria Aragón (IIS Aragón), Universidad de Zaragoza, Zaragoza, España Universidad de Zaragoza Universidad de Zaragoza Zaragoza Spain; 3 Centro de Investigación en Red de Enfermedades Cardiovasculares (CIBERCV), Instituto de Salud Carlos III, Madrid, España Instituto de Salud Carlos III Instituto de Salud Carlos III Madrid Spain; 4 Servicio de Pediatría, Hospital Universitario Miguel Servet, Zaragoza, España Hospital Universitario Miguel Servet Hospital Universitario Miguel Servet Zaragoza Spain; 5 Servicio de Bioquímica Clínica, Hospital de Alcañiz, Alcañiz, España Hospital de Alcañiz Hospital de Alcañiz Alcañiz Spain; 6 Servicio de Medicina Preventiva, Hospital Universitario Miguel Servet, Zaragoza, España Hospital Universitario Miguel Servet Hospital Universitario Miguel Servet Zaragoza Spain; 7 Centro de Salud de Barbastro, Barbastro, España Centro de Salud de Barbastro Centro de Salud de Barbastro Barbastro Spain

**Keywords:** diabetes mellitus de tipo 1, hemoglobina glicosilada, lípidos, osteocalcina, niños, Diabetes mellitus, type 1, glycated hemoglobin, lipids, osteocalcin, children

## Abstract

**Introducción.:**

La diabetes mellitus de tipo 1 se considera una de las enfermedades crónicas más frecuentes de la infancia. Es un factor de gran riesgo de desarrollar enfermedad cardiovascular temprana y afecta también la salud ósea.

**Objetivo.:**

Describir las características demográficas y los parámetros bioquímicos de una población de niños con diabetes de tipo 1, supervisados en la unidad pediátrica de diabetes de un hospital español de tercer nivel.

**Materiales y métodos.:**

En este estudio retrospectivo, se determinaron los parámetros de control metabólico, lipídico y óseo en 124 niños con diabetes de tipo 1, a los que se hizo seguimiento en la Unidad Pediátrica de Diabetes del Hospital Universitario Miguel Servet de Zaragoza, desde mayo del 2020 hasta julio del 2021.

**Resultados.:**

Los niños con diabetes de tipo 1 presentan peor control metabólico de la enfermedad en la pubertad, pero su control lipídico se puede considerar aceptable. Existe una correlación inversa de los marcadores de formación ósea con el tiempo de evolución de la enfermedad, así como con el control metabólico.

**Conclusión.:**

Los marcadores de formación ósea se encuentran correlacionados de forma inversa con el porcentaje de hemoglobina glicosilada y con el tiempo de evolución de la diabetes. En estos pacientes, el perfil lipídico y el óseo son más favorables cuando existe un buen control metabólico de la enfermedad.

## Abstract (en)

**Introduction.:**

Type 1 diabetes mellitus is considered one of the most common chronic diseases of childhood. It is a high-risk factor for developing early cardiovascular disease and it also affects bone health.

**Objective.:**

To describe demographic characteristics and biochemical parameters of a population of children with type 1 diabetes, evaluated in the pediatric diabetes unit of a tertiary Spanish hospital.

**Materials and methods.:**

In this retrospective study, we determined metabolic, lipid, and bone parameters in 124 children with type 1 diabetes who were monitored in the pediatric diabetes unit of the *Hospital Universitario Miguel Servet* in Zaragoza (Spain) from May 2020 to July 2021.

**Results.:**

Children with type 1 diabetes have worse metabolic control of the disease at puberty, but their lipid control is considered acceptable. We found an inverse correlation between bone formation markers and disease duration, as well as with metabolic control.

**Conclusion.:**

Bone formation markers are inversely correlated with the percentage of glycated hemoglobin and diabetes evolution time. Patients’ lipid and bone profiles are more favorable when metabolic control of the disease is achieved.

La diabetes mellitus de tipo 1 se considera una de las enfermedades crónicas más frecuentes de la infancia. Su incidencia varía considerablemente según la localización geográfica, la más baja en China y Sudamérica (menos de un caso por 100.000 habitantes, al año) y la más alta en Finlandia (57,6 por 100.000 habitantes, al año) ^1^. Los distintos registros prospectivos nacionales e internacionales (DIAMON, EURODIAB) han revelado un aumento progresivo de su incidencia mundial en las últimas décadas; se ha reportado un aumento anual del 2 al 5 %, el cual parece ser mayor entre los grupos de menor edad, en Europa, Australia y Medio Oriente ^2^^-^^4^.

En Hispanoamérica, existe un aumento de la diabetes infantil en los últimos años, ya que uno de cada 100 niños padece diabetes de tipo 1; la diabetes de tipo 2 también está en aumento debido a la creciente prevalencia de la obesidad infantil. Específicamente en México, más de medio millón de niños padecen diabetes de tipo 1 y se presentan casi 80.000 nuevos casos cada año. En España, la situación es similar, con un aumento constante de los casos de diabetes infantil, estimándose que más de 16.000 niños padecen diabetes de tipo 1. Según la *International Diabetes Federation*, a nivel mundial se prevé un diagnóstico anual de diabetes mellitus de tipo 1 de más de 500.000 niños por debajo de los 15 años ^5^. Por todo ello, la diabetes mellitus de tipo 1, hoy en día, representa una enfermedad de gran prevalencia en la población infantil y en la juvenil ^6^.

Respecto a las complicaciones cardiometabólicas en los niños con diabetes mellitus de tipo 1, se sabe que estos pacientes se consideran de “alto riesgo cardiovascular” ^7^^,^^8^, por lo que se han propuesto y publicado guías y protocolos para su adecuado control metabólico ^9^^,^^10^. La presencia de dislipemia en estos pacientes -caracterizada por una disminución en la concentración de colesterol de alta densidad (HDL) y el incremento de la concentración del colesterol de baja densidad (LDL) y de los triglicéridos ^11^- aumenta su riesgo cardiovascular.

Sin embargo, a pesar de la evidencia sobre la relación existente entre el grado de control glucémico y el riesgo de inicio o progresión de las complicaciones, microvasculares (retinopatía, nefropatía y neuropatía) y macrovasculares (cardiovasculares, cerebrovasculares y enfermedad vascular periférica), la mayoría de los niños y adolescentes diabéticos no presentan un control óptimo de sus niveles de glucosa ^12^^,^^13^.

Por otra parte, también es bien conocido el efecto que tienen los dos tipos de diabetes mellitus en la salud ósea, ya que alteran la cantidad y la calidad del hueso, lo que resulta en un aumento en la tasa de fracturas. El uso de técnicas avanzadas de imágenes pone de manifiesto los efectos adversos de la diabetes mellitus de tipo 1 en el desarrollo del esqueleto, los cuales se extienden más allá de la densidad mineral ósea e incluyen anormalidades en el tamaño, la forma y la microarquitectura ósea, así como en la fuerza muscular ^14^.

El metabolismo óseo de los niños se caracteriza por un crecimiento más rápido y complejo, con procesos simultáneos de crecimiento y remodelado óseo en diferentes zonas del hueso, el cual difiere del metabolismo del adulto, en el que se acoplan estrechamente los procesos de formación y resorción ósea. Por ello, la interpretación de los marcadores óseos en niños resulta más difícil, siendo necesario disponer de valores de referencia según la edad, el sexo, el estadio puberal, los tratamientos y las enfermedades asociadas con el crecimiento óseo.

Los marcadores óseos analizados en este estudio fueron osteocalcina y fosfatasa alcalina ósea, dos de los más utilizados en la práctica clínica y que permiten evaluar el proceso de formación ósea, esencial en la adquisición de una masa ósea adecuada.

El objetivo de este estudio fue describir las características demográficas y perfilar el control metabólico y la salud cardiovascular y ósea de un grupo de niños con diabetes mellitus de tipo 1 supervisados en la unidad pediátrica de diabetes del hospital de tercer nivel Miguel Servet de Zaragoza (España).

## Materiales y métodos

Se llevó a cabo un estudio descriptivo, retrospectivo, con datos de una prueba analítica anual de control realizada en la revisión rutinaria trimestral de los niños afectados por diabetes mellitus de tipo 1 que se siguen en la Unidad Pediátrica de Diabetes del Hospital Universitario Miguel Servet de Zaragoza.

En esta Unidad, se supervisa un total de 290 niños, de los cuales se analizaron 124, desde mayo del 2020 hasta julio del 2021. Se excluyeron los diagnósticos de *novo* establecidos en dicho periodo por la falta de controles de seguimiento, así como los niños con una enfermedad aguda o que estuviesen en tratamiento con alguna medicación que pudiera interferir con el metabolismo lipídico o el óseo (aunque no fue necesario excluir ningún niño por este último motivo).

El estudio se realizó de acuerdo con la Declaración de Helsinki de 1964 y sus actualizaciones posteriores, y recibió la aprobación del Comité de Ética de Investigación de la Comunidad de Aragón.

Se recogieron en la muestra datos como la puntuación z del índice de masa corporal (IMC) y el fototipo -siguiendo la escala validada de Fitzpatrick-, los años de evolución de la enfermedad, si presentaban alguna otra enfermedad autoinmunitaria asociada (tiroidea, celiaquía o enfermedad celiaca, u otras menos frecuentes) y el estadio puberal según la clasificación de Tanner.

Respecto a los parámetros analíticos, se determinaron el perfil bioquímico general y la hemoglobina glicosilada (HbA1c), así como el perfil metabólico y el óseo.

En cuanto al tratamiento recibido, se anotaron las necesidades diarias de insulina y el esquema terapéutico de los pacientes: múltiples dosis de insulina o infusión continua subcutánea de la misma.

El análisis estadístico se llevó a cabo con el programa IBM SPSS™ Statistics, versión 21.0 (IBM Corporation, Armonk, NY). Mediante la prueba de Kolmogorov-Smirnov, se comprobó el tipo de distribución que seguían las variables cuantitativas. Cuando las variables presentaban una distribución normal (p > 0,05), se describieron con la media y la desviación estándar, y se compararon con otras variables cuantitativas (de dos categorías) mediante la prueba t de Student.

Cuando las variables cuantitativas no presentaban una distribución normal (p ≤ 0,05), se describieron con la mediana y el rango intercuartílico, y para su análisis respecto a otras variables cuantitativas (de dos categorías), se aplicó la prueba U Mann-Whitney. Cuando se trataba de variables cuantitativas con más de dos categorías, se utilizó la prueba de Kruskal-Wallis con la corrección de Bonferroni.

Para el análisis entre variables cualitativas, se utilizó la prueba de ji al cuadrado (c^2^). Además, se analizó la correlación existente entre las variables cuantitativas mediante la prueba de Pearson y la de Spearman, dependiendo del tipo de distribución (normal o no). Como dato estadístico de contraste, se utilizó la prueba estadística de ji al cuadrado (c^2^). El nivel de significancia estadística se estableció a partir de un valor de probabilidad igual o menor de 0,05 (p ≤ 0,05).

## Resultados

La muestra incluyó 124 niños con una distribución homogénea entre niños (43,5 %) y niñas (56,5 %). El porcentaje de prepúberes fue del 33,1 %. Con respecto al fototipo de los pacientes de la muestra, según la escala validada de Fitzpatrick, los resultados fueron los siguientes: tipo 1: 0 %; tipo 2: 25 %; tipo 3: 54,8 %; tipo 4: 16,9 %, y tipo 5: 3,2 %.

En relación con el IMC, la mayoría de los niños se encontraba dentro de la normalidad, solo el 7,25 % presentaba un IMC > 25 y ninguno de los niños presentaba un IMC superior a 30, ni menor que el percentil 5 en la tabla de las curvas de crecimiento.

Entre las enfermedades autoinmunitarias, la tiroidea fue la de mayor frecuencia (11,3 %), seguida de la celiaquía (7,3 %) y otras, como déficit de inmunoglobulina IgA, neutropenia, psoriasis o púrpura trombocitopénica inmunológica, cuyas frecuencias fueron extremadamente bajas (0,8 % cada una de ellas).

Como tratamiento, al 78,2 % de los pacientes se les administraban la insulina en múltiples dosis y, al resto, en infusión continua. De aquellos con múltiples dosis de insulina, el 41,9 % utilizaba glargina como insulina basal, el 21 %, detemir, y el 16,1%, degludec.

Las características antropométricas del grupo, como la edad y el IMC, así como los años de evolución de la enfermedad, necesidad de insulina total y bolos de insulina rápida que precisaban los niños al día, se muestran en la tabla 1.

**Tabla 1:** Características antropométricas y tratamiento recibido por niños con diabetes mellitus de tipo 1

Variable	Niños		Niñas		Total	
Sexo (%)	43,5		56,5		100	
Edad (años)	14	(4,97)^1^	14	(7,13)^1^	13,9	(7)^1^
Evolución (años)	4	(3,99)^1^	5	(6,08)^1^	4,3	(4.81)^1^
IMC (kg/m2)	20	(3,61)^2^	19	(2,84)^2^	19,62	(3,23)^2^
Número de bolos de insulina por día	4	(1)^1^	4	(1)^1^	4	(1)^1^
Insulina (UI/kg/día)	0,98	(0,18)^1^	0,76	(0,39)^1^	0,79	(0,38)^1^

IMC: índice de masa corporal

^1^ Mediana y rango intercuartílico

^2^ Media y desviación típica

En las variables cuantitativas, se muestran las medidas de tendencia central y desviación, según los resultados de las pruebas de normalidad. La prueba de normalidad realizada fue la de Kolmogorov-Smirnov.

Los resultados analíticos de los parámetros bioquímicos generales, el control glucémico, el metabolismo óseo y el lipídico, solicitados en la prueba analítica de control anual, se muestran en las tablas 2 y 3, respectivamente.

**Tabla 2:** Parámetros del metabolismo glucídico y el óseo

Variable	Niños		Niñas		Total	
Glucosa (mg/dl)	174,74	(62,71)^2^	183,62	(71,05)^2^	178,74	(67,35)^2^
HbA1c (%)	7,8	(1,2)^1^	7,75	(1,4)^1^	7,84	(18,60)^2^
Vitamina D (ng/ml)	26,8	(7,4)^2^	23,42	(8,8)^2^	25,41	(7,44)^2^
PTHi (pg/ml)	37,67	(16,05)^1^	40,52	(17,38)^1^	26	(9,5)^1^
Calcitonina (pg/ml)	3,29	(2,04)^1^	2,65	(1,27)^1^	2	(1)^1^
Calcio (mg/dl)	9,84	(0,34)^1^	9,77	(0,29)^1^	9,90	(0,4)^1^
Fósforo (mg/dl)	4,69	(1)^2^	4,60	(1)^2^	4,65	(0,68)^2^
Fosfatasa alcalina ósea (U/L)	112,5	(76,2)^1^	90,75	(95,6)^1^	101,805	(48,56)^2^
Osteocalcina (ng/ml)	55,5	(47)^1^	48,75	(47)^1^	62,48	(47,859)^1^

HbA1c: Hemoglobina glicosilada; PTHi: hormona paratiroidea intacta

^1^ Mediana y rango intercuartílico

^2^ Media y desviación típica

Se aplicó la prueba de normalidad de Kolmogorov-Smirnov.

**Tabla 3:** Parámetros del metabolismo lipídico

Variable	Niños		Niñas		Total	
Colesterol total	168	(36)^1^	177	(35)^1^	173	(37)^1^
Colesterol HDL	58,54	(11,15)^2^	65,28	(13,06)^2^	61,58	(12,46)^2^
Colesterol LDL	98	(26)^1^	102	(28)^1^	100	(27)^1^
Triglicéridos	62,5	(28)^1^	55	(33)^1^	59	(31)^1^

HDL: lipoproteínas de alta densidad; LDL: lipoproteínas de baja densidad

^1^ Mediana y rango intercuartílico

^2^ Media y desviación típica

Se aplicó la prueba de normalidad de Kolmogorov-Smirnov.

El 20,97 % de los pacientes presentaba buen control metabólico, según las recomendaciones actuales de la *American Diabetes Association* (HbA1c < 7 %). No se encontraron diferencias en cuanto al porcentaje de HbA1c, entre niños y niñas (p = 0,301).

Al comparar las posibles diferencias existentes en los niveles de HbA1c en función del estadio puberal, considerando por separado cada categoría en la que se definía la variable (estadios I, II, III, IV y V), según la clasificación de Tanner, se encontraron diferencias significativas entre los distintos grupos. Mediante un análisis pareado, se identificó que esta diferencia estaba entre el grupo con estadio puberal V -que tiene asociado un nivel más alto de HbA1c- y los grupos I (p = 0,050) y IV (p = 0,017) (tabla 4).

**Tabla 4:** Diferencias en los niveles de HbA1c según el estadio puberal Valor K-W: resultado de la prueba de Kruskal-Wallis. Hay diferencias entre el estadio I y el V; y entre IV y el V. El grupo V tiene valores más elevados que el I y el IV

HbA1c(%)		Mediana	Rango intercuartílico	Valor mínimo	Valor máximo
Estadio I		7,4	1,3	6,1	8,9
Estadio II		7,3	1,2	6,6	8,9
Estadio III		7,5	1,7	7,1	10,0
Estadio IV		6,9	0,4	6,7	7,3
Estadio V		8,1	1,4	5,8	11,7
**Estadio puberal**			Valor K-W	p	
	Estadio IV versus V		-48,263	0,017	
	Estadio I versus V		-20,482	0,050	

No se encontraron diferencias significativas en el porcentaje de HbA1c entre quienes recibían múltiples dosis de insulina y aquellos con infusión continua subcutánea de la misma, ni entre los distintos fármacos empleados en el esquema con múltiples dosis de insulina (glargina, detemir o degludec).

Asimismo, la variable continua “años de enfermedad” se subdividió en dos categorías (0 = menos de cinco años de enfermedad, 1 = más de cinco años de enfermedad), para determinar si existían diferencias en el porcentaje de HbA1c entre ambos grupos. Se observaron diferencias significativas (p < 0,001) entre los pacientes con menos de cinco años de evolución -presentaban menor porcentaje de HbA1c- y los que llevaban más de cinco años de evolución de la enfermedad -presentaban valores más elevados de HbA1c-.

En cuanto a la concentración de lípidos, solo el 3,2 % de los pacientes presentó más de 130 mg/dl de triglicéridos y, el 12,5 %, tenía concentraciones de más de 130 mg/dl de colesterol LDL. La media del colesterol HDL fue de 61,53 mg/dl y casi todos los pacientes presentaban valores de colesterol HDL por encima de 40 mg/dl (99 %). Además, los niños con buen control metabólico (HbA1c < 7 %) tenían menor concentración de triglicéridos (U = 81; p = 0,005), al compararlos con los que tenían mal control metabólico, según la prueba U de Mann-Whitney, por tratarse de una distribución no paramétrica.

Finalmente, al estudiar la correlación de los distintos marcadores óseos con el tiempo de evolución de la enfermedad y con el porcentaje de hemoglobina glicosilada, se halló una correlación inversa entre la concentración de fosfatasa alcalina ósea y el tiempo de evolución de la enfermedad (r = -0,370; p < 0,001), y entre el valor de la HbA1c y el de la concentración de osteocalcina (r = -0,286; p = 0,001). Asimismo, existe una correlación inversa entre el porcentaje de HbA1c y la concentración de fosfatasa alcalina ósea (r = -0,345; p = 0,001), y entre el porcentaje de HbA1c y la concentración de calcitonina (r = -0,257; p = 0,006).

## Discusión

Los niños con diabetes de tipo 1 en la pubertad presentan peor control metabólico de la enfermedad, sin embargo, el control lipídico de los niños con diabetes de tipo 1 se puede considerar aceptable. Existe una correlación inversa entre los marcadores de formación ósea y el tiempo de evolución de la enfermedad. El perfil lipídico y el óseo de estos pacientes son más favorables cuando existe un buen control metabólico de la diabetes.

La muestra poblacional incluida en este estudio presenta características similares a las descritas en publicaciones anteriores, por lo que no se encontraron diferencias de incidencia por sexo, resultados coincidentes con los reportados en otras regiones del país y del mundo ^15^. La enfermedad autoinmunitaria más frecuente fue la tiroidea, seguida de la celiaquía ^16^.

En este estudio, los niños con diabetes de tipo 1 durante la pubertad tuvieron peor control metabólico de la enfermedad, ya que el grupo con estadio puberal V , se asoció con un mayor valor de HbA1c. En este sentido, en los niños adolescentes con diabetes ya se han reconocido cambios hormonales y psicológicos como causa frecuente del mal cumplimiento del tratamiento y del mal control metabólico ^17^. El control de la diabetes en la pubertad está modulado por un cambio en la síntesis y la secreción de varias hormonas, incluyendo la hormona de crecimiento, que se asocia con cambios en la sensibilidad a la insulina ^18^. Asimismo, durante la pubertad, se presenta un aumento de las necesidades de insulina y un cambio en las necesidades nutricionales, lo cual -unido a los cambios emocionales- implica una dificultad en el control de la diabetes.

El valor medio de la HbA1c encontrado coincide plenamente con el que muestran los niños en el estudio SWEET iniciado en el 2008, el cual incluía a niños diabéticos de 22 centros repartidos por todo el mundo ^19^. También, se han observado peores niveles de HbA1c conforme los niños diabéticos llevan más años de evolución de la enfermedad ^20^.

No se encontraron diferencias estadísticamente significativas en el porcentaje de HbA1c, según el tratamiento bien hubiera sido con múltiples dosis de insulina o por infusión continua subcutánea, a diferencia de Faienza *et al*. ^21^, quienes detectaron menor porcentaje de HbA1c en los niños tratados con infusión continua subcutánea que en los tratados con múltiples dosis de insulina.

La frecuencia de dislipemia (alteración en la concentración de lípidos, especialmente por encima de los valores recomendados) entre los niños diabéticos suele oscilar entre el 65 y el 75 %, según diferentes estudios ^22^^,^^23^; en el presente trabajo, el 12,5 % de los niños tenía una concentración de colesterol LDL aumentada (superior a 130 mg/dl), resultados similares a los encontrados en el estudio de Bulut *et al*. ^24^. En dicho estudio, el 15,8 % de los niños tenía concentración de colesterol LDL superior a 130 mg/dl, pero aquellos supervisados en el Hospital Miguel Servet presentaban mejor perfil lipídico al considerar la concentración de triglicéridos, puesto que solamente el 3,2 % de ellos tenía hipertrigliceridemia (concentración de triglicéridos >130 mg/dl) frente al 15,8 % del estudio de Bulut *et al*.

Sin embargo, el límite deseable de colesterol LDL en estos pacientes es mantener concentraciones iguales o inferiores a 100 mg/dl. En este sentido, en el grupo de niños del presente estudio, solamente el 51,6 % (alrededor de la mitad) tenía estas concentraciones, resultados muy parecidos a los del estudio SEARCH for *Diabetes in Youth* (SEARCH), en el cual, el 48 % de los pacientes presentó una concentración de colesterol LDL igual o inferior a 100 mg/dl ^25^. En un estudio realizado en Italia en el 2018, Macedoni *et al*. ^26^, encontraron que el 73,7 % de los pacientes estudiados tenía concentraciones de colesterol LDL iguales o inferiores a 100 mg/dl.

Por lo tanto, es necesario monitorizar de forma estricta la concentración de lípidos en estos pacientes, así como asesorarles para que mantengan una alimentación saludable y una adecuada condición física.

Cuando se consideró la concentración de lípidos en función del control metabólico, se pudo comprobar que la de triglicéridos era más elevada si existía un control metabólico inadecuado de la enfermedad. Estos resultados coinciden con los obtenidos por la mayoría de los autores en la literatura ^27^^-^^29^.

En relación con el metabolismo óseo, se determinó que existe una correlación inversa entre la concentración de fosfatasa alcalina ósea y el tiempo de evolución de la enfermedad, así como entre la concentración de calcitonina, de fosfatasa alcalina ósea y de osteocalcina, y el control metabólico (proporción de HbA1c) de los niños con diabetes.

Estos resultados son similares a los reportados por Abd-El Dayem *et al*. ^30^ y Maggio *et al*. ^31^, quienes encontraron una correlación inversa entre el porcentaje de HbA1c y la concentración de osteocalcina. En un estudio llevado a cabo por Pater *et al*. ^32^, en 2010, se puso de manifiesto que un control glucémico adecuado tiene un efecto beneficioso en los marcadores de recambio óseo, lo cual puede prevenir una masa ósea disminuida cuando los niños se conviertan en adultos.

En el metaanálisis de Madsen *et al*. del año 2019 ^33^, se evidenció que la concentración de osteocalcina (marcador de formación ósea) en los niños y adolescentes con diabetes mellitus de tipo 1, era significativamente menor que la de niños y adolescentes sanos; sin embargo, no se encontraron diferencias en los marcadores de resorción ósea (β-CrossLaps o β-CTX), por lo que se cree que existe una menor formación ósea y no una mayor resorción en estos niños. Esta menor formación ósea es la que podría dar lugar a menor densidad mineral, tal y como se sugiere en el estudio de Tsentidis *et al*. ^34^.

Sin embargo, hasta el día de hoy, todavía es objeto de controversia la existencia de una menor densidad mineral ósea en los niños con diabetes mellitus de tipo 1. Aunque en algunos estudios se ha encontrado asociación entre diabetes mellitus tipo 1 y reducción de la densidad mineral ósea ^35^, en otros ^36^^,^^37^ no se ha identificado.

Brandao *et al*. ^36^ no encontraron dicha asociación, pero reconocían que una larga duración de la enfermedad y un pobre control metabólico, podían tener un impacto negativo en la masa ósea de los niños con diabetes, probablemente reduciendo el pico de densidad mineral ósea con las consecuencias que ello conlleva, como fracturas en edades más avanzadas.

En un estudio de seguimiento llevado a cabo por Vázquez-Gámez *et al*. ^38^, se observó que los niños y adolescentes con diagnóstico reciente de diabetes mellitus de tipo 1 presentaban una densidad mineral ósea normal, pero, con el transcurso del tiempo -y especialmente durante la adolescencia- tenían menor ganancia de masa ósea, así como alteraciones en los parámetros del metabolismo óseo. Además, en un estudio llevado a cabo en mujeres con diabetes mellitus de tipo 1 en edades posteriores a la adolescencia (entre los 20 y los 37 años) pudo comprobarse una disminución de la densidad mineral ósea en la cadera, lo que puede explicar en parte la alta incidencia de fractura de cadera en mujeres posmenopáusicas con diabetes mellitus de tipo 1 ^38^.

Puesto que los cambios detectados en la concentración de marcadores de recambio óseo preceden a los cambios de la densidad mineral ósea, su determinación puede resultar de gran interés para detectar niños que puedan presentar problemas óseos. No obstante, aunque algunos estudios miden la densidad mineral ósea en los niños con diabetes mellitus de tipo 1, muy pocos reportan los marcadores de recambio óseo en niños y adolescentes con esta afección.

Por otra parte, en niños y adolescentes con diabetes mellitus de tipo 1, existe un aumento del estrés oxidativo, probablemente involucrado en el deterioro del proceso de recambio óseo que se produce en ellos ^39^. Por esta razón, la estrategia de tratamiento debe ir dirigida a mejorar el control glucémico para disminuir el estrés oxidativo, lo que redundará en una mejor prevención o tratamiento para la salud ósea de estos niños.

Como limitaciones de este estudio, se resalta el pequeño tamaño muestral y su carácter retrospectivo. Esto indica la necesidad de desarrollar estudios prospectivos que permitan establecer la relación causal entre los distintos marcadores óseos, el control metabólico, el estrés oxidativo y la disminución de la densidad mineral ósea en los niños con diabetes mellitus de tipo 1.
